# Parliament and Publications

**Published:** 1910-11-12

**Authors:** 


### Parliament and Publications.

### THE DUBLIN HOSPITALS.

The Fifty-Second Annual Report of the Board of Superintendence of the Dublin Hospitals deals with all the Dublin Hospitals which receive grants from Parliament. The report gives extracts from the latest edition of "Burdett's Hospitals and Charities," and it appears to the Board that the figures therein set forth reflect the greatest credit on the management of Dublin Hospitals. One of the many tables in the Report shows the average cost per bed occupied throughout the year ended March 31, 1910, for maintenance and for establishment, and for both, exclusive of buildings and furnishing such buildings. The table is as follows :—

| Name of Hospital. | Average Daily Number of Beds occupied throughout the Year. | Average Annual Cost per Bed for Maintenance. | | | Average Annual Cost per Bed for Establishment, exclusive of Buildings and Furnishing such Buildings. | | | Average Annual Cost per Bed for Maintenance and for Establishment, exclusive of Buildings, and Furnishing such Buildings. | | |
|---|---|---|---|---|---|---|---|---|---|---|
| | | £ | s. | d. | £ | s. | d. | £ | s. | d. |
| Westmoreland Lock ... ... | 58.64 | 13 | 9 | 1 | 29 | 15 | 4 | 43 | 4 | 5 |
| Steevens' ... ... ... | 102.89 | 23 | 7 | 5 | 43 | 17 | 4 | 67 | 4 | 9 |
| Meath ... ... ... ... | 115.17 | 20 | 14 | 11 | 36 | 7 | 5 | 57 | 2 | 4 |
| Cork Street ... ... ... | 129.43 | 14 | 10 | 10 | 40 | 2 | 1 | 54 | 12 | 11 |
| House of Industry ... ... | 160.07 | 15 | 1 | 6 | 36 | 7 | 6 | 51 | 9 | 0 |
| Rotunda Lying-in ... ... | 89.83 | 15 | 14 | 2 | 49 | 12 | 11 | 65 | 7 | 1 |
| Coombe Lying-in ... ... | 45.35 | 13 | 1 | 7 | 40 | 10 | 11 | 53 | 12 | 6 |
| Royal Victoria Eye and Ear. | 82.37 | 18 | 5 | 6 | 27 | 5 | 7 | 45 | 11 | 1 |
| Royal Hospital for Incurables | 207.33 | 12 | 7 | 4 | 24 | 13 | 1 | 37 | 0 | $5\frac{3}{4}$ |

In giving consideration to these figures it must be borne in mind (says the report) that since in-patient and out-patient departments are worked mainly by one staff it is difficult to apportion the cost of each. As a consequence the cost of the out-patient department has been ignored in arriving at the above figures, and to arrive at the average cost per bed the total cost of maintenance and establishment has been divided by the average number of beds occupied.—" Fifty-Second Annual Report of the Board of Superintendence of the Dublin Hospitals," Cd. 5335, price 3d.

### THE HEALTH OF THE METROPOLITAN POLICE.

The Report of the Chief Surgeon on the health of the Metropolitan Police Force during the year 1909 shows that the total number of sick in that year was 12,118, of whom 11,088 resumed duty, 139 were reported unfit, and 34 died. Six officers not on the sick list died suddenly. The total number of men for whom payment was made to the divisional surgeons was on June 30, 1909, 17,285, and on December 31, 1909, 17,478, giving as the mean 17,381. The average daily number of men (1) on the sick list was 454, (2) on sick leave 28, (3) on detached leave 32. This gives as the daily average loss in the whole force by sickness a percentage of 2.80, the full strength of the force being 18,657. The numbers of police known to have been admitted into the general hospitals of the Metropolis on the recommendation of the chief or divisional surgeons, and with the consent of the Commissioner, was 275; 32 were admitted into cottage hospitals, and 81 into special hospitals. Nine men were admitted to workhouse infirmaries for observation, five of whom were certified insane and sent to asylums. Fifty-four men were suspended on account of venereal disease, and 37 of these were sent to the Lock Hospital or Military Hospital, Rochester Row. It is further stated in the Report that at the end of January 1909 the Schäfer method of artificial respiration was adopted by the police. During the eleven months up to December 31, 1909, the new method was used in 31 cases, 23 of which were cases of immersion; of these 16 were successful and seven unsuccessful. In three other cases both the Schäfer and Silvester methods were used; one was successful. In one case a woman had poisoned herself with paraffin and then attempted suicide by drowning. Artificial respiration by the Schäfer method was employed and animation restored, but she died in hospital from the paraffin poisoning.—" Report of the Commissioner of Police of the Metropolis for the Year 1909," Cd. 5404, price $9\frac{1}{2}$d.

### THE REASSEMBLING OF PARLIAMENT.

Parliament meets again on Tuesday, November 15, and at the commencement of public business the Prime Minister will move that for the remainder of the Session, Government business have precedence at every sitting. Among the Government Bills down on the orders of the day for second reading is the Public Health (Health Visitors) Bill, and among the other measures down for second reading are the Public Health Act (1875) Amendment Bill and the Coroners' Law and Death Certification (Amendment) Bill. Sir Henry Craik has given notice of a blocking motion against the last-named Bill.

### RAILWAY ACCIDENTS.

The number of persons killed and injured on railways in the United Kingdom in the course of public traffic during the three months ending June 30, 1910, as reported to the Board of Trade was as follows: Passengers 15 killed, 528 injured; servants of companies or contractors, 70 killed, 1,035 injured; persons passing over railways at level crossings, 20 killed, 7 injured; trespassers (including suicides), 100 killed, 47 injured; other persons, 7 killed, 34 injured; total, 212 killed, 1,651 injured.—" Railway Accidents," Cd. 5403, price $8\frac{1}{2}$d.

### HEALTH IN REFORMATORY AND INDUSTRIAL SCHOOLS.

The general death-rate in reformatory and industrial schools in Great Britain for the year 1909 was lower than for the previous year, being 3.09, as against 3.49, and was in fact the lowest ever recorded for these schools. The rate of discharges on account of physical unfitness was also lower, being 2.58, as against 2.80 for 1908. This, in the view of the inspector, is all the more creditable to the schools, as there is a fairly general impression that the physique of the children on admission has been appreciably inferior during the last year or two as compared with former years. This is perhaps more particularly the case as regards the young offenders sent to reformatory schools. The instructor affirms that there are instances where the number of pigeon-chested, rickety boys with defective circulation and sores of various kinds admitted to the school is so great as to tempt one to say that it is only when crime is coupled with some physical weakness or defect that the culprit is deemed to be a fit subject for a reformatory."—" Fifty-third Report of the Inspector appointed to Visit Reformatory and Industrial Schools," Cd. 5406, price $6\frac{1}{2}$d.

